# Haemodynamic Monitoring in the Intensive Care Unit: Results from a Web-Based Swiss Survey

**DOI:** 10.1155/2014/129593

**Published:** 2014-04-22

**Authors:** Nils Siegenthaler, Raphael Giraud, Till Saxer, Delphine S. Courvoisier, Jacques-André Romand, Karim Bendjelid

**Affiliations:** ^1^Intensive Care Unit, Department of Anaesthesiology, Pharmacology and Intensive Care, University Hospitals of Geneva, 4 Rue Gabrielle-Perret-Gentil, 1211 Geneva 14, Switzerland; ^2^Faculty of Medicine, University of Geneva, 4 Rue Gabrielle-Perret-Gentil, 1211 Geneva 14, Switzerland; ^3^Division of Clinical Epidemiology (Biostatistics), University Hospitals of Geneva, 4 Rue Gabrielle-Perret-Gentil 1211 Geneva 14, Switzerland

## Abstract

*Background*. The aim of this survey was to describe, in a situation of growing availability of monitoring devices and parameters, the practices in haemodynamic monitoring at the bedside. *Methods*. We conducted a Web-based survey in Swiss adult ICUs (2009-2010). The questionnaire explored the kind of monitoring used and how the fluid management was addressed. *Results*. Our survey included 71% of Swiss ICUs. Echocardiography (95%), pulmonary artery catheter (PAC: 85%), and transpulmonary thermodilution (TPTD) (82%) were the most commonly used. TPTD and PAC were frequently both available, although TPTD was the preferred technique. Echocardiography was widely available (95%) but seems to be rarely performed by intensivists themselves. Guidelines for the management of fluid infusion were available in 45% of ICUs. For the prediction of fluid responsiveness, intensivists rely preferentially on dynamic indices or echocardiographic parameters, but static parameters, such as central venous pressure or pulmonary artery occlusion pressure, were still used. *Conclusions*. In most Swiss ICUs, multiple haemodynamic monitoring devices are available, although TPTD is most commonly used. Despite the usefulness of echocardiography and its large availability, it is not widely performed by Swiss intensivists themselves. Regarding fluid management, several parameters are used without a clear consensus for the optimal method.

## 1. Introduction

Adequate haemodynamic assessment and management are cornerstones for the management of critically ill patients [1, 2]. However, the use of haemodynamic monitoring at the bedside faces many challenges. First, the methods, devices, and parameters available for haemodynamic monitoring have evolved over the last 30 years, and this may be responsible for the large heterogeneity in the types of techniques used by clinicians in various intensive care units (ICUs). Second, the proper use of these monitoring devices and the interpretation of the values displayed may be difficult and require a high level of knowledge and skill, resulting in heterogeneous interventions [3, 4]. Third, advanced methods for haemodynamic monitoring, per se, have not been associated with an improvement in patient survival [5–9], unless they are coupled with early and clinically relevant therapeutic strategies [1]. Consequently, the integration of measured parameters into the therapeutic strategy may also vary between physicians and ICUs. Finally, in some situations, the macrocirculation may be decoupled from the microcirculation [10, 11], thereby reducing the effectiveness of haemodynamic optimisation based only on commonly measured macrocirculatory parameters and complicating the haemodynamic management of critically ill patients.

Considerable heterogeneity in the availability and practice of haemodynamic monitoring exists at the bedside across clinicians, ICUs, and countries, although studies investigating this issue are scarce [3, 12–15]. However, this type of study could allow for tailored training in intensive care and could help to adapt the clinical guidelines according to the techniques available. The goal of this study was, thus, to generate a first description of the availability and the use of bedside haemodynamic monitoring in Swiss ICUs, especially for the management of volume expansion.

## 2. Methods

This study was designed as a self-reported, internet-based survey. The questionnaire consisted of 36 multiple-choice questions (http://www.genevahemodynamic.com/research/swisshaemodynamicsurvey). Apart from general questions the questions investigated two topics: the monitoring techniques used by Swiss intensivists (16 questions) and the method by which Swiss intensivists address fluid management (8 questions). Advanced haemodynamic monitoring was defined as the use of techniques that allow the estimation of cardiac output. In questions reporting frequency of use, clinicians rate their utilisation on a scale from 1 to 10 (1 = never, 10 = in every case). In questions qualifying a device, clinicians were asked to scale their replies from 0 to 5 (0 = “the worst,” 5 = “the best”). The questionnaire was first evaluated by two independent physicians specialised in critical haemodynamic care and then tested on 15 Swiss intensivists to improve the formulation of the questions.

We selected all adult ICUs (medical, surgical, and interdisciplinary) that conform to the recommendation of the Swiss Society of Intensive Medicine 2008-2009 (77 ICUs). We sent the questionnaire via e-mail to the physician responsible for the selected ICUs and/or to physicians working in the same centre that could be identified. The contacted physician could then decide to reply and/or to forward the questionnaire to some of his colleagues in the same ICU. To increase the return rate, the questionnaire was sent a second time to nonresponders. Replies were collected during the period from 2009 to 2010. As this survey was based on voluntary participation with an information disclosure, an ethics committee did not review this study.

### 2.1. Statistical Analysis

Data were analysed using R 2.14.1. We analysed the responses either at the physician level or the ICU level. Responses analysed at the physician level consider each physician's answer as having equivalent weight. Thus, ICUs with more responding physicians contributed more responses. To give equal weight to all ICUs, we also calculated the responses at the ICU level by determining the opinion of each centre, corresponding to the majority of replies in the centre, and then averaging the opinion of all ICUs. To determine the contribution of the number of replies per ICU to the results, we analysed the correlation between the responses averaged across ICUs versus that averaged across individual physicians. Regarding the description of replies concerning parameters that require a specific technique (e.g., extravascular lung water (EVLW), which can only be measured with the PiCCO device (PULSION Medical systems; Munich, Germany)), we selected only those replies from physicians working in ICUs where this device was available. To evaluate the degree of consensus for each question, we arbitrarily determined that a response rate greater than 65% for a single question represented a strong consensus, a response rate between 55 and 64% indicated a weak consensus, and a response rate less than 55% represented no consensus. For multiple-choice questions, a positive consensus was reached if the physicians who participated included the proposition, and a negative consensus was reached if the physicians who participated did not include the proposition.

## 3. Results

### 3.1. Descriptive Analysis

We obtained 130 replies from 55 ICUs (71.4%) from a total of 77 Swiss adult ICUs referenced during the study period. The median response rate was 1 per ICU (1–20 replies per ICU; mean response rate: 2.3; interquartile range: 1). Among the participating intensivists, 73% (*n* (Intensivists) = 95/130) declared to be specialists in intensive care medicine (certification from the Swiss Medical Association). In addition, 62% (*n* (Intensivists) = 81/130) reported more than 5 years of experience in critical care practice (5–10 years: 25% (*n* (Intensivists) = 33/130), >10 years: 37% (*n* (Intensivists) = 48/130)). The correlation between the replies reported by individual physicians and by ICUs was very high (*r* = 0.997, *P* < 0.0001), suggesting that the response rate of individual centres (i.e., the “size” of the ICU) did not influence the results.

### 3.2. Availability and Use of Haemodynamic Monitoring in Swiss ICUs

**Figure 1:**
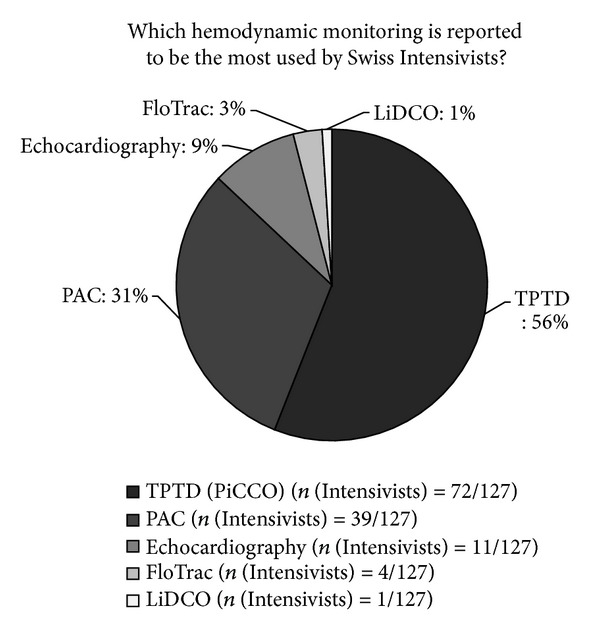
Haemodynamic monitoring techniques reported to be most commonly used by intensive care physicians. TPTD: transpulmonary thermodilution, PAC: pulmonary artery catheter. The results are presented as the mean number of replies from Swiss intensivists (in %) to the total number of intensivists who replied to the question (*n*(Intensivists)/total replies).

In Switzerland, intensivists reported frequent use of advanced haemodynamic monitoring during the shock state; for example, during cardiogenic and septic shock, the mean rate of use was 8.3/10 and 8.1/10, respectively. Three devices were most commonly available: echocardiography (95% (*n* (ICU) = 52/55)), right heart thermodilution with pulmonary artery catheter (PAC: 85% (*n* (ICU) = 47/55)), and transpulmonary thermodilution (TPTD) with the PiCCO device (82% (*n* (ICU) = 45/55)). FloTrac, oesophageal Doppler monitoring, and LiDCO were not widely available (20% (*n* (ICU) = 11/55), 13% (*n* (ICU) = 7/55), and 9% (*n* (ICU) = 5/55), resp.). Notably, in 67% (*n* (ICU) = 37/55) of Swiss ICUs, TPTD and PAC were both available, although TPTD was reported to be more commonly used (Figure 1). In ICUs where PAC was reported to be most frequently used, 78% (*n* (ICU) = 7/9) were leading centres recommended for critical care teaching (Swiss Medical Association class A ICUs).

**Figure 2:**
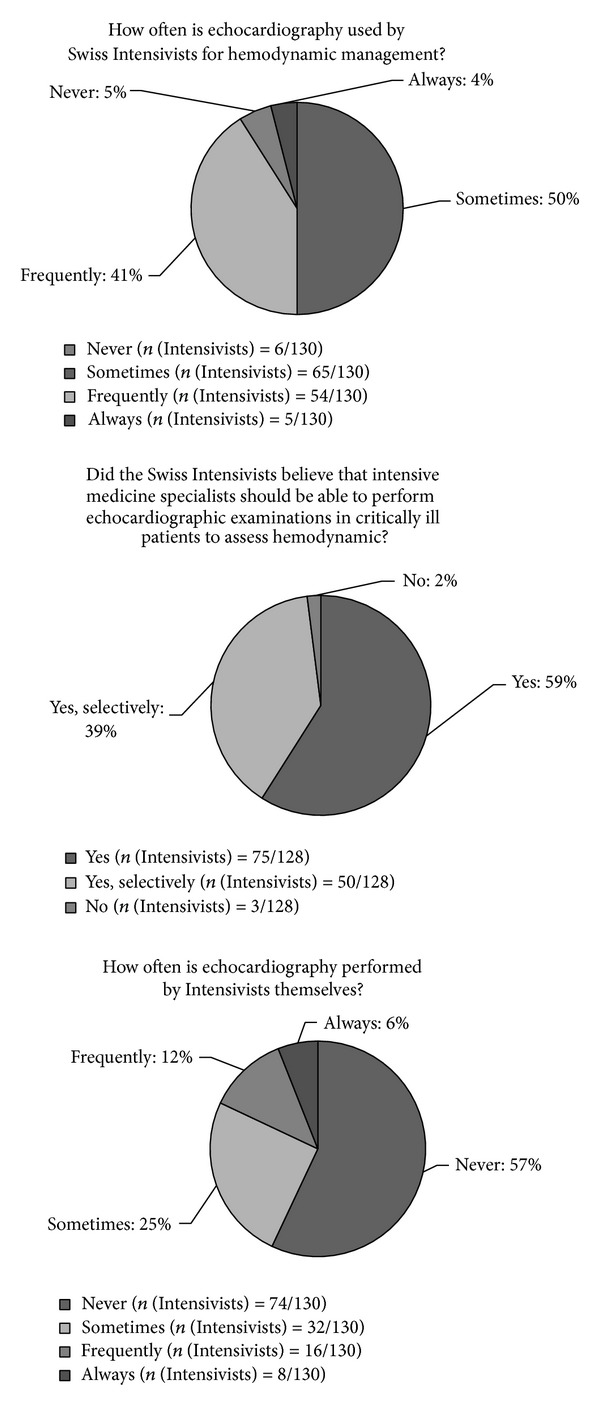
The use of echocardiography by intensivists. The results are presented as the mean number of replies from Swiss intensivists (in %) to the total number of intensivists who replied to the question (*n*(Intensivists)/total replies).

Echocardiography was available in most ICUs (Figure 2) but was not routinely used, and in most cases, echocardiography was not performed by the intensivists themselves. In contrast to this result, a large majority of participating physicians considered that Swiss intensivists should be able to perform echocardiography in ICUs for haemodynamic management.

### 3.3. Clinically Oriented Selection of Haemodynamic Monitoring

**Figure 3:**
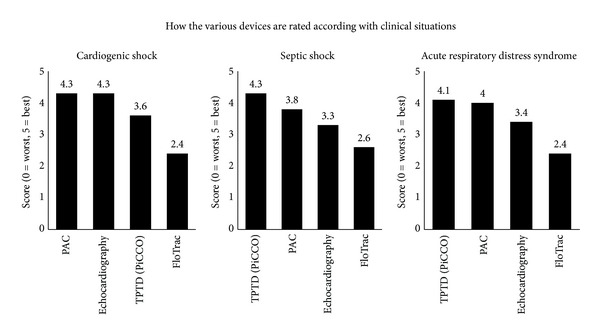
Evaluation of various devices by intensivists according to the clinical situation. Devices were rated on a scale from 1 “worst” to 5 “best.” TPTD: transpulmonary thermodilution, PAC: pulmonary artery catheter.

The method considered optimal for haemodynamic monitoring varied according to the clinical situation (Figure 3). During cardiogenic shock, Swiss intensivists considered monitoring with PAC or echocardiography equally good and reported these two monitoring techniques superior to other techniques. During septic shock, intensivists considered TPTD to be the most appropriate monitoring technique. Finally, during acute respiratory distress syndrome (ARDS), intensivists considered TPTD and PAC to be the best techniques; interestingly, these two techniques were considered to be equivalent in this situation.

### 3.4. Parameters Used with TPTD and the PiCCO Device

**Figure 4:**
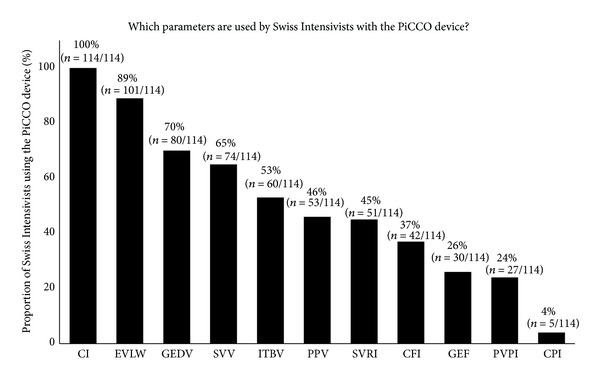
The use of various parameters available with transpulmonary thermodilution (PiCCO) by Swiss intensivists. CFI: cardiac function index; CI: cardiac index; CPI: cardiac power index; EVLW: extravascular lung water; GEDV: global end-diastolic volume; GEF: global ejection fraction; ITBV: intrathoracic blood volume; PPV: pulse pressure variation; PVPI: pulmonary vascular permeability index; SVRI: systemic vascular resistance index; SVV: stroke volume variation. The results of this multiple-choice question are presented as the mean number of replies from Swiss intensivists (in %) to the total number of intensivists who replied to the question (*n*(Intensivists)/total replies).

Among all parameters associated with the PiCCO device, only cardiac index, EVLW, global end-diastolic volume (GEDV), stroke volume variation (SVV), and intrathoracic blood volume (ITBV) were used by a majority of clinicians (Figure 4).

### 3.5. Haemodynamic Parameters Used by Swiss Intensivists for Fluid Management

For the management of fluid therapy, guidelines were available in less than half of ICUs (45%, *n* (ICU) = 25/55). The mean arterial blood pressure targeted by the majority of intensivists was between 60 and 65 mmHg (40–50 mmHg: 2% (*n* (Intensivists) = 3/130), 50–55 mmHg: 2% (*n* (Intensivists) = 3/130), 55–60 mmHg: 8% (*n* (Intensivists) = 10/130), 60–65 mmHg: 56% (*n* (Intensivists) = 73/130), and 65–70 mmHg: 27% (*n* (Intensivists) = 35/130) and 70–75 mmHg: 5% (*n* (Intensivists) = 6/130)). For the prediction of fluid responsiveness (Table 1), Swiss intensivists mainly used dynamic indices (i.e., indices which vary with respiration, e.g., pulse pressure variation, PPV), the passive leg rising manoeuvre (PLR), and/or echocardiographic parameters. Static parameters (i.e., parameters which did not varies with respiration) such as central venous pressure (CVP) and pulmonary artery occlusion pressure (PAOP) were also used by a significant number of intensivists (Table 1); however, when these methods were used, most intensivists considered that only low values indicated a state of preload dependency (CVP < 5 mmHg: 42% (*n* (Intensivists) = 55/130), CVP < 10 mmHg: 19% (*n* (Intensivists) = 25/130), CVP < 15 mmHg: 2% (*n* (Intensivists) = 2/130), and none: 37% (*n* (Intensivists) = 48/130); PAOP < 5 mmHg: 21% (*n* (Intensivists) = 24/114), PAOP < 10 mmHg: 31% (*n*(Intensivists) = 35/114), and PAOP < 15 mmHg: 21% (*n* (Intensivists) = 24/114), PAOP < 20 mmHg: 3% (*n* (Intensivists) = 3/114) and None: 25% (*n* (Intensivists) = 28/114)). On the other hand, to assess the possibility of further fluid filling, intensivists use different parameters, mainly EVLW and PAOP, according to the technique available (TPTD versus PAC).

### 3.6. Evaluation of Consensus

The results are displayed in Table 2.

## 4. Discussion

The present self-reported internet-based survey investigated the types of haemodynamic monitoring available in ICUs of a European country and reported how this monitoring is used at the bedside. We observed that, in Swiss ICUs, advanced haemodynamic monitoring is frequently used at the bedside. Among the techniques accessible, echocardiography, TPTD, and/or PAC were largely available in most ICUs; moreover, in a large majority of ICUs, both PAC and TPTD with PiCCO were available, but TPTD seemed to be the most frequently used technique. Echocardiography was largely available and considered a good technique in various situations, although this examination is generally not performed by intensivists themselves. Finally, for assessing fluid responsiveness, intensivists seemed to prefer dynamic indices instead of static parameters (Tables 1 and 2).

Limited data exists concerning the use of haemodynamic monitoring in critically ill patients at bedside across countries, but, as suggested by Torgersen et al., there seems to be considerable heterogeneity in the management and in the use of haemodynamic monitoring across centres and countries [15]. In our study, we observed a large utilisation of invasive haemodynamic monitoring in patients with shock. This practice is in accordance with the acknowledged importance of early and adequate haemodynamic optimisation in critically ill patients with shock [16]. In other European countries, during septic shock, Torgersen et al. reported that almost all responders asserted the cardiac output, even if the rate of invasive haemodynamic monitoring use was not reported. The fact that, in our country, echocardiography monitoring is less used may explain the higher rate of invasive technique observed in the present survey. Also, we may speculate that the skill and medical education of clinicians as well as the hospitals resources have a big influence on the way the critically ill patients are monitored.

The availability in a single centre of several types of haemodynamic monitoring techniques may allow the clinician, taking into account the specificities of each technique, to adapt the monitoring used in accordance with the clinical situation. However, as suggested by numerous previous studies [3, 14, 17], this implies the need for major training to ensure the proper use of different techniques and the adequate interpretation of measured parameters to correctly guide therapeutic interventions. Our observation of a large use of diverse techniques suggests that it may be interesting to assess the clinical and cost effectiveness of each technique in the management of critically ill patients. If further studies confirmed the availability of multiple devices in each ICU, a national program for teaching, maintenance of skills and regular evaluation of knowledge could be implemented to optimise the resources needed and maintain a high quality of use of these specific techniques. Indeed, in Switzerland there are no clear guidelines, specific recommendation, or nationally structured formation about the haemodynamic monitoring of patients in shock state. And the absence of any consensus on this issue makes the Swiss intensivist clinical practice associated to the local medical tradition.

Furthermore, as observed in other studies [9, 15], we noticed in our study that intensivists seemed to favour the use of new monitoring devices, such as TPTD with PiCCO, instead of the “historical” PAC method. The only exception concerned the leading centres involved in critical care teaching (Swiss Medical Association class A ICUs), where PAC remains largely used. Interestingly, in our study TPTD with PiCCO is considered by intensivists to be equivalent to PAC during ARDS and superior during septic shock, whereas during cardiogenic shock, PAC and echocardiography are considered the most appropriate techniques. Our results regarding the use of TPTD with PiCCO during septic shock are in accordance with the typical practice in European countries, in which most clinicians (65.5%) report the use of TPTD for the measurement of cardiac output in this situation [15]. Our observation of the clinical preference to use PAC or echocardiography during cardiogenic shock seems also in accordance with the study by Trof et al. comparing volume-limited (monitored by TPTD) versus pressure-limited (monitored by PAC) haemodynamic management in septic and nonseptic shock [18]. In this study, the authors did not observe any difference in ventilators-free days, lengths of stay, organ failures, and mortality between the two modes of haemodynamic monitoring. However, in the nonseptic shock patients, TPTD based algorithm (EVLW < 10 mL/kg, GEDV < 850 mL/m^2^) resulted in more days on mechanical ventilation and ICU length of stay compared with PAC (PAOP < 18–20 mmHg).

Interestingly in our study, during ARDS the monitoring with TPTD (PiCCO) is considered, by the clinicians, to be equivalent with the monitoring with PAC. This observation may likely represent one of the characteristics of the evolution in haemodynamic monitoring in critically ill patients. Traditionally, during ARDS, PAC has demonstrated certain advantages. First, the measurement of PAOP allows the exclusion of left ventricular dysfunction (PAOP of less than 18 mmHg), a criterion required for the diagnosis and definition of ARDS [19]. Second, PAC allows the evaluation of pulmonary artery hypertension, associated with the development of right ventricular failure [20], and enables the adjustment of pulmonary vasodilators (e.g., inhaled nitric oxide). Thus, during ARDS, other techniques such as echocardiography must be combined with TPTD to assess right ventricular function and pulmonary circulation. However, during ARDS, monitoring with TPTD may have benefits. For example, EVLW that was indexed to predicted body weight [21, 22] may allow a more precise evaluation of lung oedema than chest radiograph, where the presence of a bilateral infiltrate, which can be related to other diseases besides pulmonary oedema, may be difficult to identify. Moreover, EVLW may also be considered as a means to manage fluid balance during ARDS [21].

Echocardiography is a noninvasive advanced haemodynamic technique useful in the management of critically ill patients [23, 24]. In our study, we observed that even if echocardiography was widely available and considered to be reliable (Figure 3), this technique was not regularly used by intensivists themselves. This observation suggests that echocardiography is performed mainly by cardiologists in specific situations rather than as a true technique of haemodynamic monitoring used to regularly assess the evolution of the patient and the effect of treatment. However, we observed that a large majority of intensivists (98%) demonstrated a desire to become more independent in the practice of echocardiography in the critical care setting. This situation may be specific to countries where no specific echocardiographic training is intended for intensivists and where no specific descriptions of the skills required to practice this examination are accepted, as it is the case in Switzerland. In response to this situation, according to the will of clinicians and following the evolution of education and training in other European countries [24], the number of certified technicians and improved descriptions of the skills required to practice echocardiography in Swiss ICUs are growing [25, 26].

Among the difficulties associated with the use of haemodynamic monitoring, individual differences in the interpretation of parameters and related interventions could be significant. Apart from a high level of training, this issue may be improved by the implementation of clinical guidelines. However, as highlighted by the present survey, in most ICUs, guidelines for fluid resuscitation are not available. This underutilisation of guidelines during fluid resuscitation likely reflects the complexity of this issue and the lack of consensus on validated indices available to adequately predict fluid responsiveness in the large population of critically ill patients. Indeed, we failed to detect a strong consensus on the use of these indices among Swiss intensivists, although we did observe some consensus related to the nonuse of various parameters (Table 2). In the assessment of preload dependency, a slight majority of intensivists reported to use mainly dynamic indices (PPV), volumetric indices estimated with the TPTD technique (GEDV), or echocardiography, although a strong consensus was lacking. Notably, despite the amount of clinical data supporting the uselessness of static parameters (CVP, PAOP) as markers of fluid responsiveness [27, 28], a significant proportion of intensivists still use these static indices. Our reported utilisation of PAOP is comparable to that of other European countries, where 28.3% of clinicians still use PAOP to guide haemodynamic management during septic shock [15]. However, it should be noted that when these static measurements are used, intensivists consider only low values as a sign of hypovolemia-preload dependency, although there is no consensus as to the precise threshold. Similarly, to evaluate the safety of infusing further fluid, a slight majority of intensivists reported to use EVLW or PAOP, according to the technique available, as techniques to interrupt volume expansion, again without a consensus as to the preferred technique.

### 4.1. Limitations

First, it was not possible to determine the exact number of intensivists working in Switzerland and therefore to determine the true significance of our results. However, the response rate from all Swiss ICUs concerning the present survey was high, with the majority of responders experienced in intensive care medicine. Secondly, haemodynamic monitoring requires devices, accessories, consumables, and staff education that have financial implications. Indeed, economic characteristics of the institution and health economics of the country may influence the practice at bedside. Third, even if this survey is related to the 2009-2010 period, we consider that our results represent the actual evolution in the practice of haemodynamic monitoring at the bedside, as no major changes in hemodynamic monitoring practice and guidelines occur recently. Fourth, in order to describe the degree of consensus or agreement about the practice of haemodynamic monitoring, we used a simple method which, even if not well validated, allows to identify the “general opinion” of clinicians. Lastly, as demonstrated by a previous study [29], the difference between the perception of a practice and the real life practice at the bedside may be significant. Thus, our results are only indicative of self-reported practice in haemodynamic monitoring and only further prospective observational studies will be able to more precisely investigate this subject.

### 4.2. Conclusion

In our survey of haemodynamic monitoring in Swiss ICUs, we found that various types of monitoring techniques are available in ICUs, among which the “historical” PAC method seems to be progressively replaced by new monitoring techniques, such as TPTD.

As an alternative or complementary technique, echocardiography, which is largely available in Swiss ICUs, was not frequently used by intensivists themselves to regularly assess the haemodynamic state of critically ill patients. Concerning the utilisation of haemodynamic monitoring to guide the complex management of fluid therapy, clinical guidelines are underutilised and intensivists inconsistently refer essentially to dynamic indices of preload.

## Figures and Tables

**Table 1:** Haemodynamic parameters used by Swiss intensivists for fluid management.

Parameters	Average of replies by Swiss intensivists
*Parameters used to predict fluid responsiveness *	
PPV	59% (*n* = 76/130)
PLR	54% (*n* = 70/130)
Echocardiography	54% (*n* = 70/130)
SVV	48% (*n* = 62/130)
GEDV**	46% (*n* = 51/112)
CO	45% (*n* = 59/130)
ScvO_2_	43% (*n* = 56/130)
Arterial pressure	42% (*n* = 54/130)
PAOP*	39% (*n* = 44/114)
EVLW**	33% (*n* = 37/111)
SvO_2_*	32% (*n* = 36/113)
CVP	31% (*n* = 40/130)
RVVC	26% (*n* = 34/130)
ITBV**	21% (*n* = 24/112)
Global fluid balance	15% (*n* = 19/130)
Diameter of inferior vena cava	12% (*n* = 15/130)

*Parameters used to stop further fluid infusion *	
EVLW**	52% (*n* = 58/112)
PAOP*	51% (*n* = 58/114)
PPV	43% (*n* = 55/129)
GEDV**	42% (*n* = 47/112)
Lactate	42% (*n* = 54/129)
Echocardiography	38% (*n* = 49/128)
PLR	38% (*n* = 49/129)
ITBV**	30% (*n* = 34/112)
Other clinical parameters	27% (*n* = 35/129)
Oxygen requirement	26% (*n* = 33/129)
Normal CO	23% (*n* = 30/129)
ScvO_2_	19% (*n* = 24/129)
SvO_2_*	13% (*n* = 15/113)
High CO	6% (*n* = 8/129)

The results are presented as the mean response from Swiss intensivists in %, with the number of replies to the total number of intensivists responding to the question (*n *Intensivists/total replies). For parameters requiring a specific technique, only the replies from ICUs where this technique was available were selected: pulmonary artery catheter (PAC) available: indicated by*; transpulmonary thermodilution with PiCCO available: indicated by**. CO: cardiac output; CVP: central venous pressure; EVLW: extravascular lung water; GEDV: global end-diastolic volume; ITBV: intrathoracic blood volume; PAOP: pulmonary artery occlusion pressure; PLR: passive leg rising test; PPV: pulse pressure variation; RVVC: respiratory variation of inferior vena cava; ScvO_2_: central venous blood saturation; SVV: stroke volume variation; SvO_2_: mixed venous blood saturation.

**Table 2:** Consensus in the replies from Swiss intensivists concerning haemodynamic monitoring.

*Strong consensus *
On the availability of echocardiography, pulmonary artery catheter, or PiCCO in Swiss ICUs
On the nonavailability of FloTrac, oesophageal Doppler monitoring, or LiDCO in Swiss ICUs
On the use of echocardiography for haemodynamic monitoring
On the interest of Swiss intensivists to be able to perform echocardiography themselves in critically ill patients
On the use of cardiac index, EVLW, GEDV, or SVV when using the PiCCO device
On the nonuse of GEF, PVPI, or CPI when using the PiCCO device
On the nonuse of EVLW, SVO_2_, CVP, RVVC, ITBV, global fluid balance, or the diameter of inferior vena cava for predicting fluid
responsiveness
On the nonuse of ITBV, other clinical parameters, oxygen requirement, normal cardiac output, ScVO_2_, SVO_2_, or high cardiac output to
stop further fluid infusion

*Weak consensus *
On the preference for the use of TPTD in haemodynamic monitoring
That Swiss intensivists do not perform themselves echocardiography
On the use of ITBV when using the PiCCO device
On the nonuse of CFI when using the PiCCO device
For a mean arterial blood pressure target between 60–65 mmHg
On the use of PPV for predicting fluid responsiveness
On the nonuse of cardiac output, ScVO_2_, arterial pressure, or PAOP to predict fluid responsiveness

*No consensus *
On the frequency of use of echocardiography for haemodynamic monitoring
On the use of PPV or SVRI when using the PiCCO device
On the threshold of CVP that may indicate the need for fluid infusion
On the threshold of PAOP that may indicate the need for fluid infusion
On the use of PLR, echocardiography, SVV, or GEDV for predicting fluid responsiveness
On the use of EVLW or PAOP to stop further fluid infusion

A strong consensus was defined as a response rate greater than 65% for a single question; a weak consensus was defined as a response rate from 55–64%; and no consensus was declared when the response rate was under 55%. CVP: central venous pressure; EVLW: extravascular lung water; GEDV: global end-diastolic volume; ITBV: intrathoracic blood volume; PAOP: pulmonary artery occlusion pressure; PLR: passive leg rising test; PPV: pulse pressure variation; RVVC: respiratory variation of inferior vena cava; ScvO_2_: central venous blood saturation; SVV: stroke volume variation; SvO_2_: mixed venous blood saturation.
